# Supplementary material for: Metasurface array for single-shot spectroscopic ellipsometry
Source: Light Sci Appl. 2024 Apr 10;13:88. doi: 10.1038/s41377-024-01396-3 (PMC11006928; doi:10.1038/s41377-024-01396-3)
Supplement: Supplementary file 1 — Supplementary Information [file 41377_2024_1396_MOESM1_ESM.docx]

**Supplementary Information for**

**Metasurface array for single-shot spectroscopic ellipsometry**

Shun Wen^1,#^, Xinyuan Xue^1,#^, Shuai Wang,^1^ Yibo Ni,^1^ Liqun Sun^1^, Yuanmu Yang^1^*

^1^State Key Laboratory of Precision Measurement Technology and Instruments, Department of Precision Instrument, Tsinghua University, Beijing 100084, China

#These authors contributed equally.

*[ymyang@tsinghua.edu.cn](mailto:ymyang@tsinghua.edu.cn)

**1. Measurement of the full Stokes polarization spectrum**

For polarized light impinging on the metasurface array, it can be expressed in the form of the full Stokes polarization spectrum as $\text{S}$(*λ*) = [*s*_0_(*λ*), *s*_1_(*λ*), *s*_2_(*λ*), *s*_3_(*λ*)]^T^, where *s*_0_(*λ*), *s*_1_(*λ*), *s*_2_(*λ*) and *s*_3_(*λ*) are functions of the wavelength *λ*. The metasurface array consists of *N* elements and the polarization-dependent transmittance at each spectral channel of each metasurface element can be described by a 4 × 4 Mueller matrix (***M***). The transmitted light of the metasurface array can be expressed as $\text{S}_{\text{out}}\left( \text{λ} \right)\text{ }\text{=}\left[ \text{s}_{\text{out0}}\left( \text{λ} \right)\text{, }\text{s}_{\text{out1}}\left( \text{λ} \right)\text{, }\text{s}_{\text{out2}}\left( \text{λ} \right)\text{, }\text{s}_{\text{out3}}\left( \text{λ} \right) \right]^{\text{T}}$, where $\text{s}_{\text{out0}}\left( \text{λ} \right)\text{, }\text{s}_{\text{out1}}\left( \text{λ} \right)\text{, }\text{s}_{\text{out2}}\left( \text{λ} \right)\text{ and }\text{s}_{\text{out3}}\left( \text{λ} \right)$ are functions of the wavelength *λ*. The full Stokes polarization at *i*-th spectral channel of the transmitted light for *j*-th metasurface element can be written as,

$$\begin{aligned} \text{S}_{\text{out}}^{\text{j}}\left( \text{λ}_{\text{i}} \right)\text{ = }\left[ \begin{matrix} \text{s}_{\text{out0}}^{\text{j}}\left( \text{λ}_{\text{i}} \right) \\ \text{s}_{\text{out1}}^{\text{j}}\left( \text{λ}_{\text{i}} \right) \\ \begin{matrix} \text{s}_{\text{out2}}^{\text{j}}\left( \text{λ}_{\text{i}} \right) \\ \text{s}_{\text{out3}}^{\text{j}}\left( \text{λ}_{\text{i}} \right) \end{matrix} \end{matrix} \right]\text{ = }\text{M}^{\text{j}}\left( \text{λ}_{\text{i}} \right)\text{S}\left( \text{λ}_{\text{i}} \right)\text{ =}\text{ }\left[ \begin{matrix} \begin{matrix} \text{m}_{\text{00}}^{\text{j}}\left( \text{λ}_{\text{i}} \right) & \text{m}_{\text{01}}^{\text{j}}\left( \text{λ}_{\text{i}} \right) \\ \text{m}_{\text{10}}^{\text{j}}\left( \text{λ}_{\text{i}} \right) & \text{m}_{\text{11}}^{\text{j}}\left( \text{λ}_{\text{i}} \right) \end{matrix} & \begin{matrix} \text{m}_{\text{02}}^{\text{j}}\left( \text{λ}_{\text{i}} \right) & \text{m}_{\text{03}}^{\text{j}}\left( \text{λ}_{\text{i}} \right) \\ \text{m}_{\text{12}}^{\text{j}}\left( \text{λ}_{\text{i}} \right) & \text{m}_{\text{13}}^{\text{j}}\left( \text{λ}_{\text{i}} \right) \end{matrix} \\ \begin{matrix} \text{m}_{\text{20}}^{\text{j}}\left( \text{λ}_{\text{i}} \right) & \text{m}_{\text{21}}^{\text{j}}\left( \text{λ}_{\text{i}} \right) \\ \text{m}_{\text{30}}^{\text{j}}\left( \text{λ}_{\text{i}} \right) & \text{m}_{\text{31}}^{\text{j}}\left( \text{λ}_{\text{i}} \right) \end{matrix} & \begin{matrix} \text{m}_{\text{22}}^{\text{j}}\left( \text{λ}_{\text{i}} \right) & \text{m}_{\text{23}}^{\text{j}}\left( \text{λ}_{\text{i}} \right) \\ \text{m}_{\text{32}}^{\text{j}}\left( \text{λ}_{\text{i}} \right) & \text{m}_{\text{33}}^{\text{j}}\left( \text{λ}_{\text{i}} \right) \end{matrix} \end{matrix} \right]\left[ \begin{matrix} \text{s}_{\text{0}}\left( \text{λ}_{\text{i}} \right) \\ \text{s}_{\text{1}}\left( \text{λ}_{\text{i}} \right) \\ \begin{matrix} \text{s}_{\text{2}}\left( \text{λ}_{\text{i}} \right) \\ \text{s}_{\text{3}}\left( \text{λ}_{\text{i}} \right) \end{matrix} \end{matrix} \right]\boldsymbol{\#}\left( \text{S1} \right) \end{aligned}$$

Since the CMOS sensor can only record the light intensity, which corresponds to the element $\text{s}_{\text{out0}}\left( \text{λ} \right)$ in $\text{S}_{\text{out}}\left( \text{λ} \right)$, the transmitted light of the *j*-th metasurface element with its intensity recorded by the CMOS sensor can be represented by the first element $\text{s}_{\text{out0}}^{\text{j}}$ of the Stokes vector as,

$$\begin{aligned} \text{I}_{\text{out}}^{\text{j}}\text{ =}\sum_{\text{i} = 1}^{\text{l}} \text{s}_{\text{out0}}^{\text{j}}(\text{λ}_{\text{i}})\text{ = }\sum_{\text{i} = 1}^{\text{l}} \text{M}_{\text{0}}^{\text{j}}\left( \text{λ}_{\text{i}} \right)\text{S}\left( \text{λ}_{\text{i}} \right)\#\left( \text{S}\text{2} \right) \end{aligned}$$

where *l* is the number of spectral channels; $\text{M}_{\text{0}}^{\text{j}}\left( \text{λ}_{\text{i}} \right)$ = $\left[ \text{m}_{\text{01}}^{\text{j}}\left( \text{λ}_{\text{i}} \right)\text{, }\text{m}_{\text{02}}^{\text{j}}\left( \text{λ}_{\text{i}} \right)\text{,}\text{m}_{\text{03}}^{\text{j}}\left( \text{λ}_{\text{i}} \right)\text{, }\text{m}_{\text{04}}^{\text{j}}\left( \text{λ}_{\text{i}} \right) \right]$ is the first row of $\text{M}^{\text{j}}$($\text{λ}_{\text{i}}$). The transmitted light of the metasurface array with its intensity recorded by the CMOS sensor can be expressed as:

$$\begin{aligned} I_{\text{out}} \text{ =}\sum_{\text{i=}\text{1}}^{\text{l}} \text{M}_{\text{0}}\left( \text{λ}_{i} \right)\text{ ∙ }\text{S}\left( \text{λ}_{i} \right)\text{= }\text{M}_{\text{0}}\text{ ∙ }\text{S} \#\left( \text{S3} \right) \end{aligned}$$

where $I$_out_ is an *N* × 1 vector; $\text{M}_{\text{0}} \text{= }\left[ \text{m}_{\text{00}}\left( \text{λ}_{\text{1}} \right)\text{, }\text{⋯}\text{, }\text{m}_{\text{00}}\left( \text{λ}_{\text{l}} \right)\text{, }\text{m}_{\text{01}}\left( \text{λ}_{\text{1}} \right)\text{, }\text{⋯}\text{, }\text{m}_{\text{01}}\left( \text{λ}_{\text{l}} \right)\text{, }\text{m}_{\text{02}}\left( \text{λ}_{\text{1}} \right)\text{, }\text{⋯}\text{, }\text{m}_{\text{02}}\left( \text{λ}_{\text{l}} \right)\text{, }\text{m}_{\text{03}}\left( \text{λ}_{\text{1}} \right)\text{, }\text{⋯}\text{,}\text{m}_{\text{03}}\left( \text{λ}_{\text{l}} \right) \right]$ is an *N* × 4*l* matrix; $\text{S} \text{= }\left[ \text{s}_{\text{0}}\left( \text{λ}_{\text{1}} \right)\text{, }\text{⋯}\text{, }\text{s}_{\text{0}}\left( \text{λ}_{\text{l}} \right)\text{, }\text{s}_{\text{1}}\left( \text{λ}_{\text{1}} \right)\text{, }\text{⋯}\text{, }\text{s}_{\text{1}}\left( \text{λ}_{\text{l}} \right)\text{, }\text{s}_{\text{2}}\left( \text{λ}_{\text{1}} \right)\text{, }\text{⋯}\text{, }\text{s}_{\text{2}}\left( \text{λ}_{\text{l}} \right)\text{, }\text{s}_{\text{3}}\left( \text{λ}_{\text{1}} \right)\text{, }\text{⋯}\text{,}\text{ }\text{s}_{\text{3}}\left( \text{λ}_{\text{l}} \right) \right]^{\text{T}}$ is a 4*l* × 1 vector.

**2. Derivation of ellipsometry parameters from the measured Stokes vector**

Light reflected from a thin film can be represented by the Jones vector, as schematically shown in Fig. S1a, as,

$$\begin{aligned} \left[ \begin{aligned} {\text{E}^{\text{'}}}_{\text{p}} \\ {\text{E}^{\text{'}}}_{\text{s}} \end{aligned} \right]\text{ = }\left[ \begin{aligned} \text{R}_{\text{p}}\text{E}_{\text{p}} \\ \text{R}_{\text{s}}\text{E}_{\text{s}} \end{aligned} \right]\text{ = }\left[ \begin{aligned} {\text{R}_{\text{p}}\left| \text{E}_{\text{p}} \right|\text{e}}^{\text{iϕ}} \\ \text{R}_{\text{s}}\left| \text{E}_{\text{s}} \right| \end{aligned} \right]\#\left( \text{S4} \right) \end{aligned}$$

where $\left[ \begin{aligned} \text{E}_{\text{p}} \\ \text{E}_{\text{s}} \end{aligned} \right]$, $\left[ \begin{aligned} {\text{E}^{\text{'}}}_{\text{p}} \\ {\text{E}^{\text{'}}}_{\text{s}} \end{aligned} \right]$ is the Jones vector of the incident and reflected light, respectively; *R*_p_ and *R*_s_ are the complex reflection coefficients of the thin film for *p*- and *s*-polarized light, respectively; and $\text{ϕ}$ is the phase difference between the *p*- and *s*-polarized light.

The Stokes vector of light reflected by the thin film can be derived from Eq. (S4) as,

$$\begin{aligned} \left[ \begin{matrix} \text{s}_{\text{0}} \\ \text{s}_{\text{1}} \\ \begin{matrix} \text{s}_{\text{2}} \\ \text{s}_{\text{3}} \end{matrix} \end{matrix} \right]\text{ = }\left[ \begin{matrix} \left| \text{R}_{\text{p}}\text{E}_{\text{p}} \right|^{\text{2}}\text{ }\text{+}\left| \text{R}_{\text{s}}\text{E}_{\text{s}} \right|^{\text{2}} \\ \left| \text{R}_{\text{p}}\text{E}_{\text{p}} \right|^{\text{2}} \text{-}\text{ }\left| \text{R}_{\text{s}}\text{E}_{\text{s}} \right|^{\text{2}} \\ \begin{matrix} \text{2}\text{R}_{\text{p}}\text{R}_{\text{s}}\left| \text{E}_{\text{p}} \right|\left| \text{E}_{\text{s}} \right|\text{cos(}\text{ϕ}\text{)} \\ \text{2}\text{R}_{\text{p}}\text{R}_{\text{s}}\left| \text{E}_{\text{p}} \right|\left| \text{E}_{\text{s}} \right|\text{sin(}\text{ϕ}\text{)} \end{matrix} \end{matrix} \right]\text{ = }\left[ \begin{matrix} \left| \text{R}_{\text{p}}\text{E}_{\text{p}} \right|^{\text{2}}\text{ }\text{+}\left| \text{R}_{\text{s}}\text{E}_{\text{s}} \right|^{\text{2}} \\ \left| \text{R}_{\text{p}}\text{E}_{\text{p}} \right|^{\text{2}} \text{-}\left| \text{R}_{\text{s}}\text{E}_{\text{s}} \right|^{\text{2}} \\ \begin{matrix} \text{2}\left| \text{R}_{\text{p}} \right|\left| \text{R}_{\text{s}} \right|\left| \text{E}_{\text{p}} \right|\left| \text{E}_{\text{s}} \right|\cos\left( \text{ϕ}\text{ }\text{+}\text{ }\text{Δ} \right) \\ \text{2}\left| \text{R}_{\text{p}} \right|\left| \text{R}_{\text{s}} \right|\left| \text{E}_{\text{p}} \right|\left| \text{E}_{\text{s}} \right|\sin\left( \text{ϕ}\text{ }\text{+}\text{ }\text{Δ} \right) \end{matrix} \end{matrix} \right]\#\left( \text{S5} \right) \end{aligned}$$

where *Δ* is the phase difference of *R*_p_ and *R*_s_.

We set the incident light to linearly polarized at 45° in the experiment. Therefore, *ϕ* = 0 and $\text{E}_{\text{p}}\text{ }\text{=}\text{ }\text{E}_{\text{s}}$. Combining Eq. (S5) with Eq. (1) in the main text, one can derive that,

$$\begin{aligned} \frac{\text{s}_{\text{0}}}{\text{s}_{\text{1}}}\text{ = }\frac{\left| \text{R}_{\text{p}} \right|^{\text{2}}\text{ }\text{+}\left| \text{R}_{\text{s}} \right|^{\text{2}}}{\left| \text{R}_{\text{p}} \right|^{\text{2}} \text{-}\left| \text{R}_{\text{s}} \right|^{\text{2}}}\text{ = }\frac{{\text{[tan(}\text{Ψ}\text{)]}}^{\text{2}}\text{ }\text{+ 1}}{{\text{[tan(}\text{Ψ}\text{)]}}^{\text{2}} \text{- 1}}\text{ = -}\frac{\text{1}}{\text{cos(2}\text{Ψ}\text{)}}\#\left( \text{S6} \right) \end{aligned}$$

$$\begin{aligned} \frac{\text{s}_{\text{2}}}{\text{s}_{\text{3}}}\text{ = }\frac{\text{cos(}\text{Δ}\text{)}}{\text{sin(}\text{Δ}\text{)}}\text{ = }\frac{\text{1}}{\text{tan(}\text{Δ}\text{)}}\#\left( \text{S7} \right) \end{aligned}$$

Consequently, the ellipsometry parameters can be written as,

$$\begin{aligned} \text{Ψ}\text{ = }\frac{\text{1}}{\text{2}}\arccos\left( \text{-}\frac{\text{s}_{\text{1}}}{\text{s}_{\text{0}}} \right)\#\text{(S8)} \end{aligned}$$

$$\begin{aligned} \text{Δ}\text{ =}\arctan\left( \frac{\text{s}_{\text{3}}}{\text{s}_{\text{2}}} \right)\#\left( \text{S9} \right) \end{aligned}$$

**3. Derivation of the theoretical ellipsometry parameter**

According to the Fresnel equations, *R*_p_ and *R*_s_ can be expressed as,

$$\begin{aligned} \text{R}_{\text{p}}\text{ = }\frac{\text{r}_{\text{01}}^{\text{p}}\text{ }\text{+}\text{ }\text{r}_{\text{12}}^{\text{p}}\text{e}^{\text{-}\text{i}\text{2}\text{δ}_{\text{p}}}}{\text{1}\text{ }\text{+}\text{ }\text{r}_{\text{12}}^{\text{p}}\text{ }\text{·}\text{ }\text{r}_{\text{01}}^{\text{p}}\text{e}^{\text{-}\text{i}\text{2}\text{δ}_{\text{p}}}}\text{, }\text{R}_{\text{s}}\text{ = }\frac{\text{r}_{\text{01}}^{\text{s}}\text{ }\text{+}\text{ }\text{r}_{\text{12}}^{\text{s}}\text{e}^{\text{-}\text{i}\text{2}\text{δ}_{\text{s}}}}{\text{1}\text{ }\text{+}{\text{ }\text{r}}_{\text{12}}^{\text{s}}\text{ }\text{·}\text{ }\text{r}_{\text{01}}^{\text{s}}\text{e}^{\text{-}\text{i}\text{2}\text{δ}_{\text{s}}}}\#\left( \text{S10} \right) \end{aligned}$$

where *r*_01_ and *r*_12_ is the reflectance ratio of the interface; *δ*_p_ and *δ*_s_ are the phase change of reflected *p*- and *s*-polarized light, respectively. For a single-layer thin film, as shown in Fig. S1b, *r*_01_, *r*_12_, *δ*_p_ and *δ*_s_ can be expressed as,

$$\begin{aligned} \text{r}_{\text{01}}^{\text{p}}\text{ = }\frac{\text{n}_{\text{1}}\cos\text{θ}_{\text{0}}\text{- }\text{n}_{\text{0}}\cos\text{θ}_{\text{1}}}{\text{n}_{\text{1}}\cos\text{θ}_{\text{0}}\text{ + }\text{n}_{\text{0}}\cos\text{θ}_{\text{1}}}\text{, }\text{r}_{\text{12}}^{\text{p}}\text{ = }\frac{\text{n}_{\text{2}}\cos\text{θ}_{\text{1}}\text{- }\text{n}_{\text{1}}\cos\text{θ}_{\text{2}}}{\text{n}_{\text{2}}\cos\text{θ}_{\text{1}}\text{ + }\text{n}_{\text{1}}\cos\text{θ}_{\text{2}}}\#\left( \text{S11} \right) \end{aligned}$$

$$\begin{aligned} \text{r}_{\text{01}}^{\text{s}}\text{ = }\frac{\text{n}_{\text{0}}\cos\text{θ}_{\text{0}}\text{-}{\text{ }\text{n}}_{\text{1}}\cos\text{θ}_{\text{1}}}{\text{n}_{\text{0}}\cos\text{θ}_{\text{0}}\text{ }\text{+}\text{ }\text{n}_{\text{1}}\cos\text{θ}_{\text{1}}}\text{, }\text{r}_{\text{12}}^{\text{s}}\text{ = }\frac{\text{n}_{\text{1}}\cos\text{θ}_{\text{1}}\text{-}\text{ }\text{n}_{\text{2}}\cos\text{θ}_{\text{2}}}{\text{n}_{\text{1}}\cos\text{θ}_{\text{1}}\text{ }\text{+}\text{ }\text{n}_{\text{2}}\cos\text{θ}_{\text{2}}}\#\left( \text{S12} \right) \end{aligned}$$

$$\begin{aligned} \text{δ}_{\text{p}}\text{ = }\text{δ}_{\text{s}}\text{ }\text{ = 2π}\left( \frac{\text{d}}{\text{λ}} \right)\text{n}_{\text{1}}\cos\left\{ \text{sin}^{\text{-1}}\left[ \frac{\text{n}_{\text{2}}}{\text{n}_{\text{1}}}\sin\left( \text{θ}_{\text{2}} \right) \right] \right\}\#\left( \text{S13} \right) \end{aligned}$$

According to Eqs. (S10) - (S13), *R*_p_ and *R*_s_ are related to the film thickness *d* and refractive index *n*. Therefore, the thickness and refractive index can be obtained by calculating the reflectance ratio as,

$$\begin{aligned} \text{ρ}\text{ = }\frac{\text{R}_{\text{p}}}{\text{R}_{\text{s}}}\text{ = tan(}\text{Ψ}\text{)}\text{e}^{\text{iΔ}}\#\left( \text{S14} \right) \end{aligned}$$

where *Ψ* and *Δ* are the ellipsometry parameters.

**
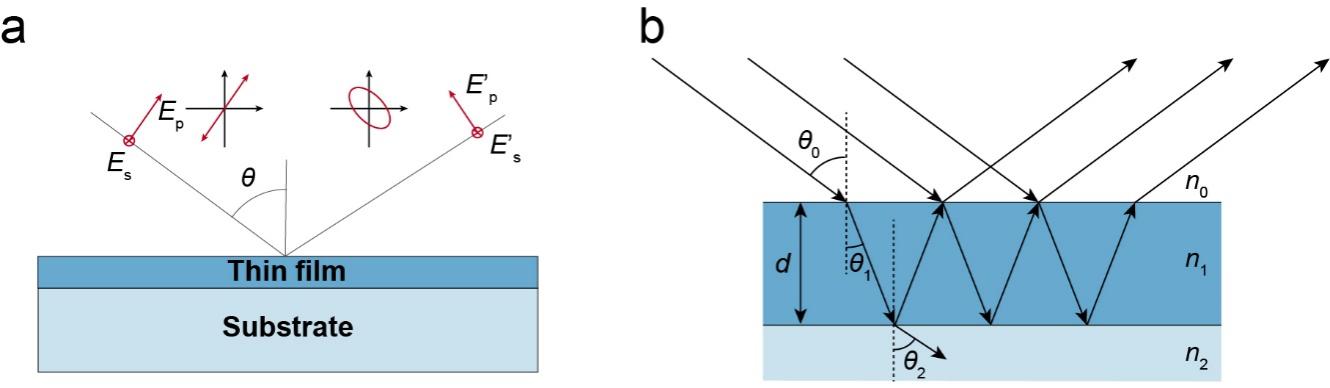
**

**Figure S1 | a,** The linearly polarized incident light is converted to an unknown (typically elliptic) polarization state upon reflection from the thin film under test. **b,** Schematic diagram of the multi-beam interference model for a single-layer thin film.

**4. Method for designing metasurface elements**

To obtain metasurface elements with anisotropic and diverse spectral features for the robust reconstruction of the full Stokes polarization spectrum, we used Lumerical FDTD^1^ to establish a database with 9000 elements. Initially, we tried to use only one set of meta-atoms^2^ based on two rotated silicon nanobricks, as shown in Fig.S2a, and found that the spectral and polarization diversity of the generated metasurface elements was not enough for the robust reconstruction of the full Stokes polarization spectrum. Hence, we added a second set of meta-atoms^3^ composed of the L-shaped nanobrick and the I-shaped nanobrick, as depicted in Fig.S2b, to allow more design freedom. The optical constants of crystalline silicon (c-Si) and sapphire (Al_2_O_3_) used in the simulation are from Palik’s handbook^4^. We changed the periodicity and the geometric parameters of the meta-atoms and established a dataset. In the dataset, there are more than 3000 elements for the first meta-atoms and more than 6000 elements for the second meta-atoms. Here, we select 400 elements from the database, with 104 elements for the first set of meta-atoms and 296 elements for the second set of meta-atoms, respectively. Complete geometric parameters of all 400 optimized metasurface elements are shown in dataset S1.


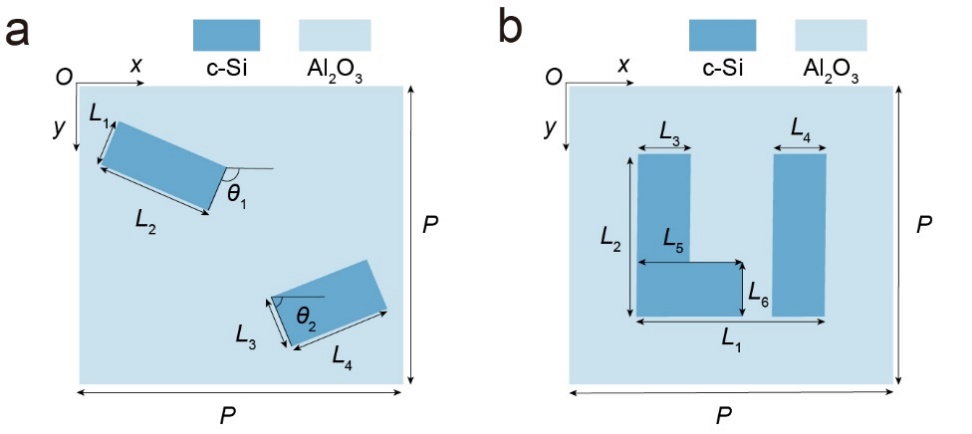


**Figure S2 | Schematic diagram of two sets of the 300 nm high crystalline silicon (c-Si) meta-atoms deposited on sapphire substrate and the definition of geometric parameters.** **a**, In the first set of meta-atoms, the rotation angles are fixed as *θ*_1_ = 112.5°, *θ*_2_ = 67.5°, respectively. The geometric centers of the two rectangles are placed at the coordinate (*P*/4, *P*/4), (3*P*/4, 3*P*/4), respectively, with the top-left corner of the meta-atom defined as the origin. The length and width of the two rectangles *L*_1_ - *L*_4_ and the periodicity *P* of the meta-atom are used as scanning parameters to generate the diverse spectropolarimetric response. **b**, In the second set of meta-atoms, the center of the pattern composed of the L-shaped nanobrick and the I-shaped nanobrick is placed at the coordinate (*P*/2, *P*/2), with the top-left corner of the meta-atom defined as the origin. The length and width of the two nanobricks *L*_1_ – *L*_6_ and the periodicity *P* of the meta-atom are used as optimization parameters to generate the diverse spectropolarimetric response. The optical constants of crystalline silicon (c-Si) and sapphire (Al_2_O_3_) used in the simulation are from Palik’s handbook ^4^.

To ensure the fidelity of full Stokes polarization spectrum reconstruction, the correlation coefficients of each row of the Mueller matrix should be minimized. The correlation coefficient is defined as,

$$\begin{aligned} \text{c}_{\text{i}\text{,}\text{ }\text{j}}\text{ }\text{=}\text{ }\frac{\text{cov}\left( \text{M}_{\text{0}}^{\text{i}}\left( \text{λ} \right)\text{, }\text{M}_{\text{0}}^{\text{j}}\left( \text{λ} \right) \right)}{\text{σ}_{\text{M}_{\text{0}}^{\text{i}}\left( \text{λ} \right)}\text{σ}_{\text{M}_{\text{0}}^{\text{j}}\left( \text{λ} \right)}}\#\left( \text{S15} \right) \end{aligned}$$

where $\text{c}_{\text{i}\text{, }\text{j}}$ is the correlation coefficient between $\text{M}_{\text{0}}^{\text{i}}\left( \text{λ} \right)$ and $\text{M}_{\text{0}}^{\text{j}}\text{(}\text{λ}\text{)}$; *i* and *j* represent transmittance responses of the *i*-th and *j*-th metasurface elements, respectively. cov and *σ* represent the covariance operation and the standard deviation, respectively.

To select an array of 20 × 20 elements, two elements with the lowest correlation coefficient are first selected as the starting point. Subsequently, the correlation coefficients between all the remaining elements and the initial two elements are calculated. The element with the smallest average correlation coefficient is chosen as the third element. This process is repeated iteratively until all 20 × 20 elements are obtained. The correlation coefficient of the finally obtained ***M***_0_(*λ*) is ***C***_0_ = [***C***_00_, ***C***_01_, ***C***_02_, ***C***_03_], which corresponds to the average correlation coefficient of [***m***_00_, ***m***_01_, ***m***_02_, ***m***_03_], as shown in Fig. S3.


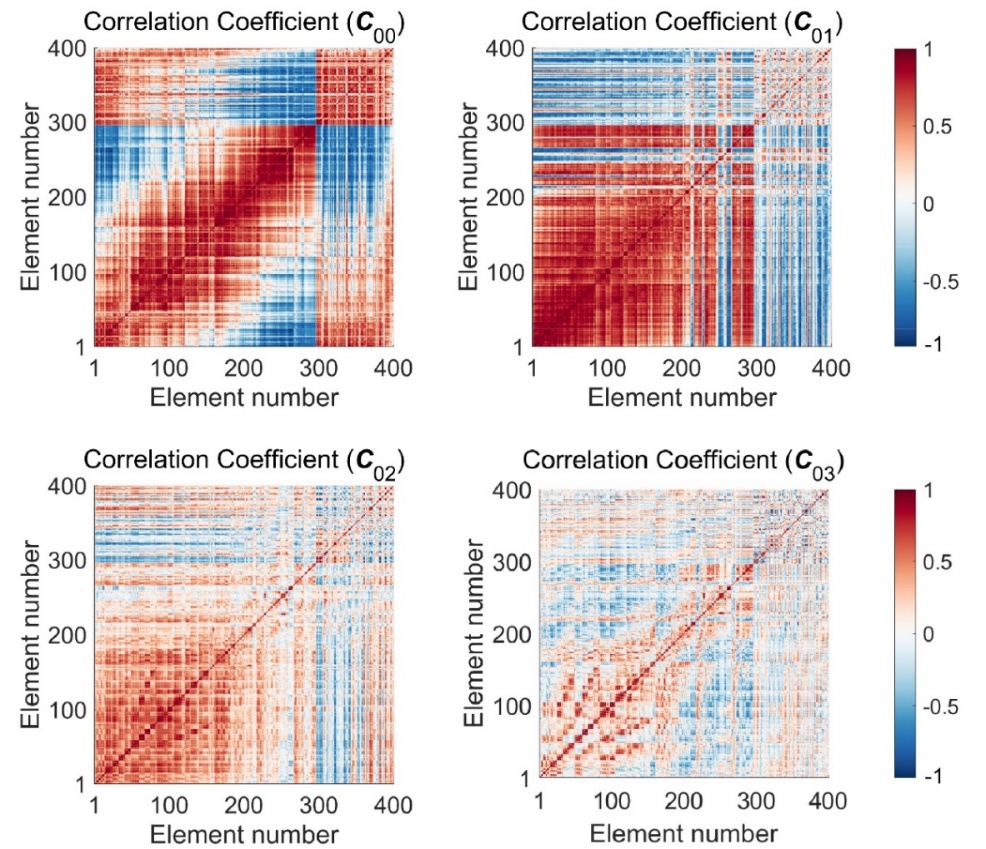


**Figure S3 |** Calculated correlation coefficients ***C***_00_, ***C***_01_, ***C***_02_ and ***C***_03_ of the first row of the Mueller matrix over different metasurface elements.

To show the reasonability of this design method, we plot the spectropolarimetric response of the first set of meta-atoms as a function of *L*_1_ - *L*_4_ and *P* in Fig. S4-S8, from which one may observe the variation of the structural parameters can indeed create a diverse spectropolarimetric response, despite that it is difficult to predict the variation trend analytically. Note that since the parameter space is too large, the plotted results in Fig. S4-S8 are just a few representative examples.


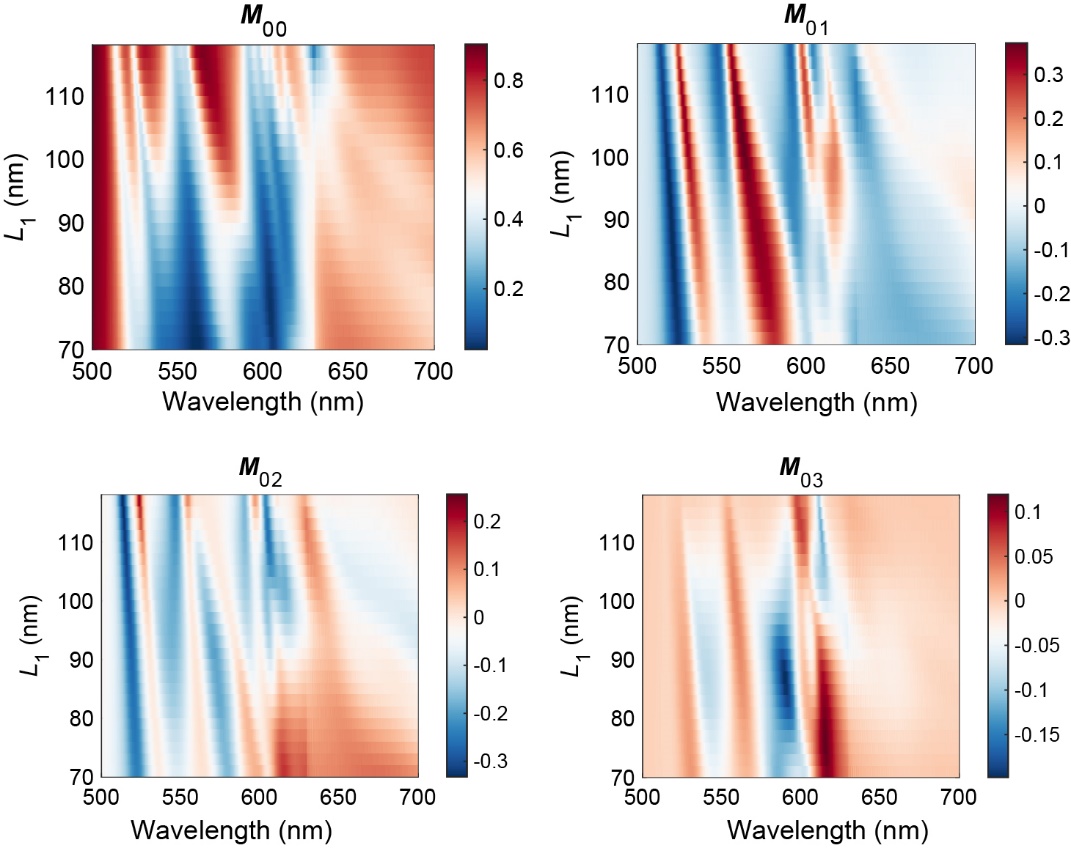


**Figure S4 |** The spectropolarimetric response of the first set of meta-atoms as a function of *L*_1_. In this plot, *L*_2_ = 130 nm, *L*_3_ = 100 nm, *L*_4_ = 140 nm, and *P =* 340 nm. ***M*** = [***M***_00_, ***M***_01_, ***M***_02_, ***M***_03_] is the Mueller matrix.


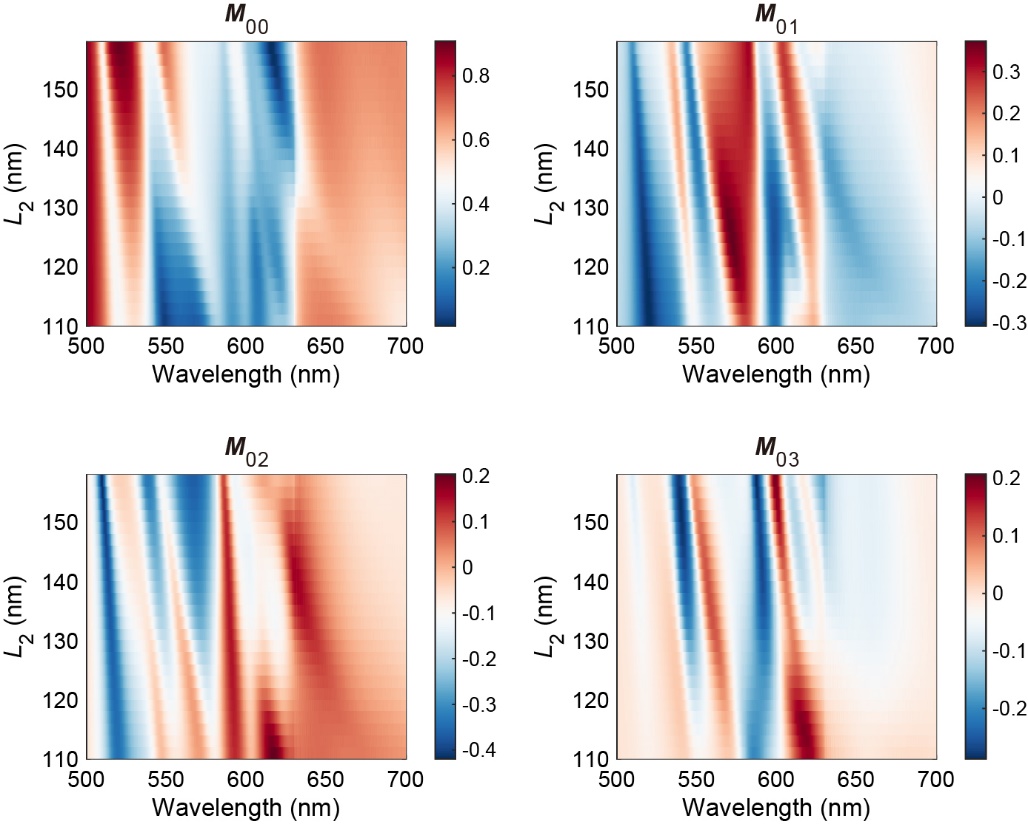


**Figure S5 |** The spectropolarimetric response of the first set of meta-atoms as a function of *L*_2_. In this plot, *L*_1_ = 80 nm, *L*_3_ = 120 nm, *L*_4_ = 160 nm, and *P =* 340 nm. ***M*** = [***M***_00_, ***M***_01_, ***M***_02_, ***M***_03_] is the Mueller matrix.


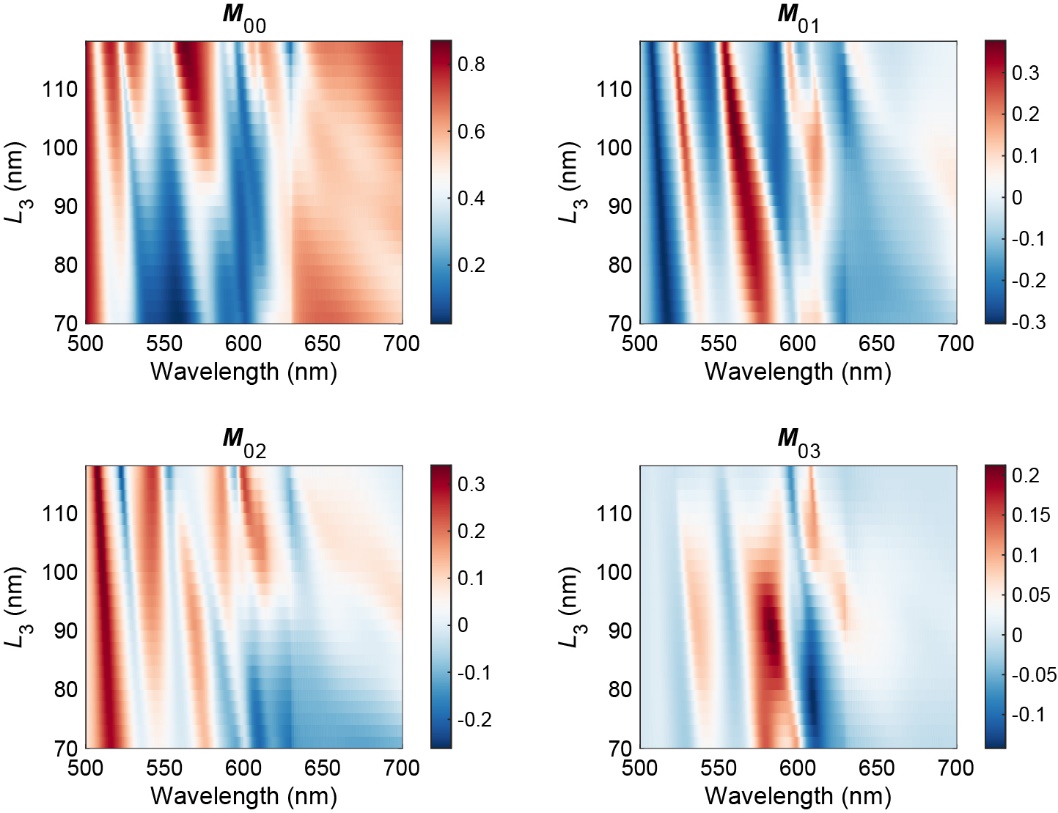


**Figure S6 |** The spectropolarimetric response of the first set of meta-atoms as a function of *L*_3_. In this plot, *L*_1_ = 130 nm, *L*_2_ = 160 nm, *L*_4_ = 120 nm, and *P =* 340 nm. ***M*** = [***M***_00_, ***M***_01_, ***M***_02_, ***M***_03_] is the Mueller matrix.


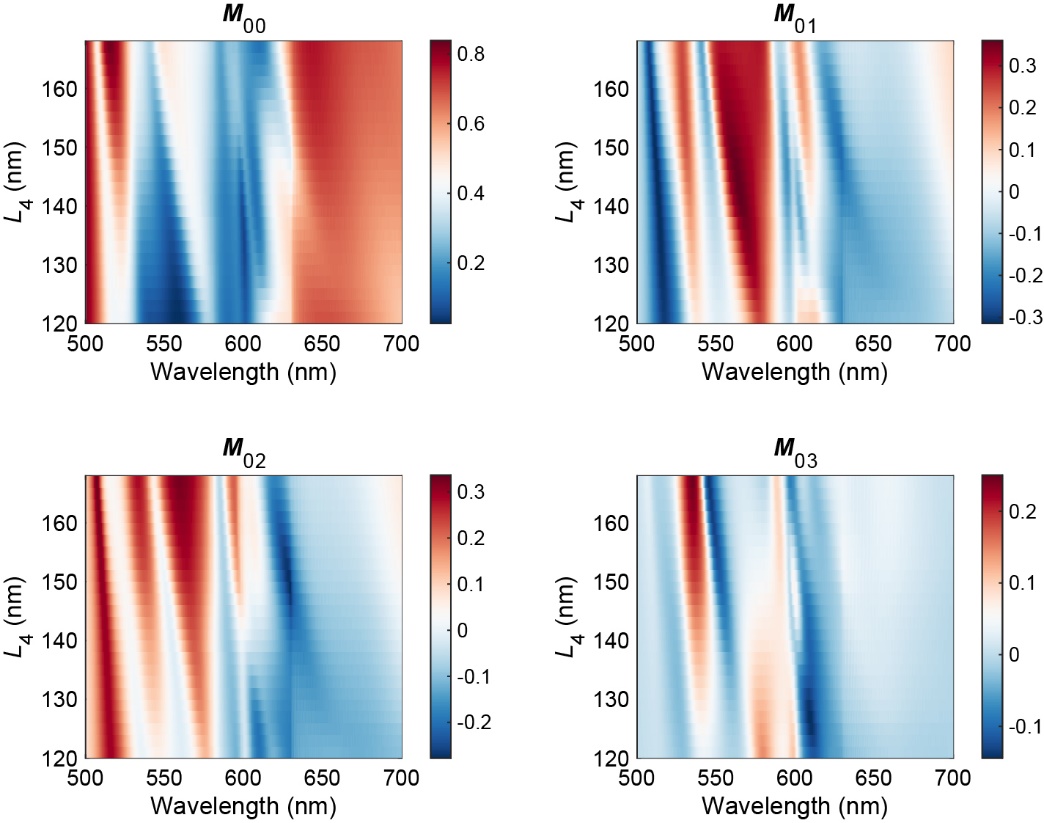


**Figure S7 |** The spectropolarimetric response of the first set of meta-atoms as a function of *L*_4_. In this plot, *L*_1_ = 130 nm, *L*_2_ = 160 nm, *L*_3_ = 70 nm, and *P =* 340 nm. ***M*** = [***M***_00_, ***M***_01_, ***M***_02_, ***M***_03_] is the Mueller matrix.


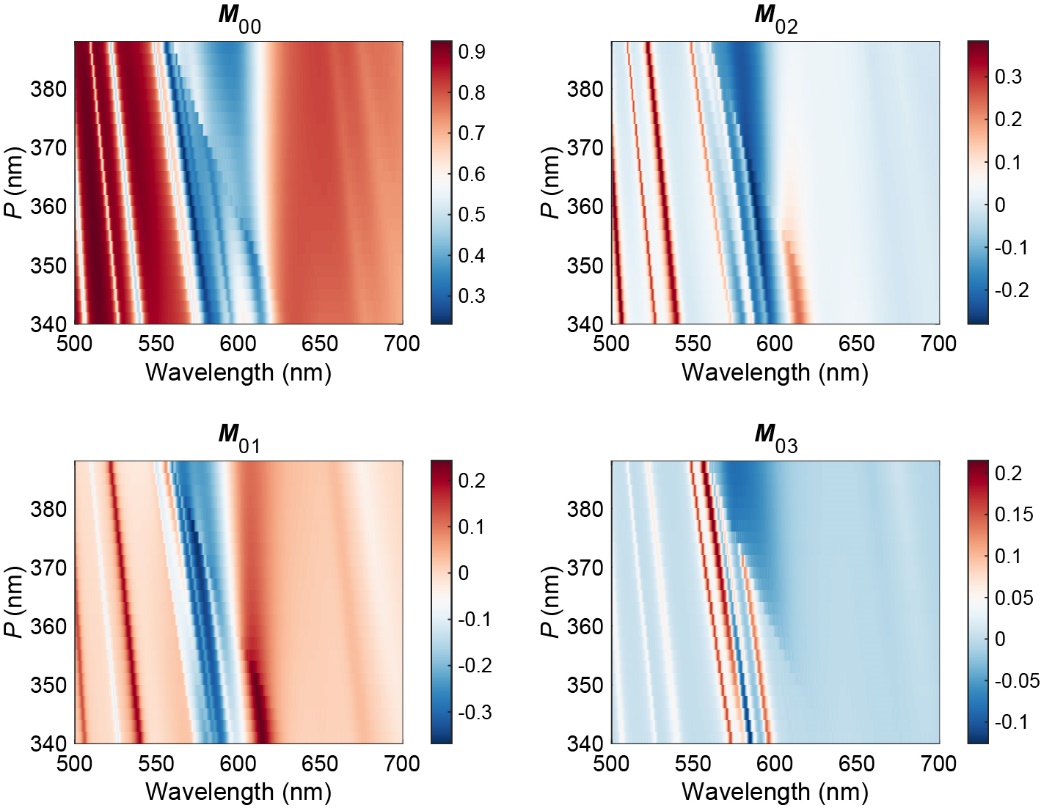


**Figure S8 |** The spectropolarimetric response of the first set of meta-atoms as a function of *P*. In this plot, *L*_1_ = 140 nm, *L*_2_ = 160 nm, *L*_3_ = 120 nm, and *L*_4_ *=* 160 nm. ***M*** = [***M***_00_, ***M***_01_, ***M***_02_, ***M***_03_] is the Mueller matrix.

To further reduce correlation in the Mueller matrix and further improved spectropolarimetric reconstruction performance, it is possible to use more complex structures, such as three angled nanobricks or two nanobricks with varying rotation angles. However, the complex structures with more optimized parameters would take additional computational resources. As a reference, in our current work, creating a database with over 9000 elements (3000 elements for two angled nanobricks, and 6000 elements for LI-shaped nanobricks) took several weeks of simulation efforts. To solve this problem, we expect that the metasurfaces with freeform shapes, combined with inverse designs, may further accelerate the design process and further improve the spectropolarimetric reconstruction performance.

**5. Fabrication tolerance for metasurface array**

To analyze the system's tolerance to fabrication errors, we simulated the Mueller matrix of a metasurface array with the optimized parameters of the meta-atoms (*L*_1_ – *L*_4_ and *P* for the first set of meta-atoms and *L*_1_ – *L*_6_ and *P* for the second set of meta-atoms) in Fig. S2 deviate from the ideal case by ±5 nm and ±10 nm, respectively. This tolerance range takes into account that the minimum gap of L-shaped nanobrick in the second set of meta-atoms is 40 nm. A larger deviation will lead to the overlap between the L-shaped nanobrick and the I-shaped nanobrick. With the fabrication errors, if the post-processing algorithm does not realize the Mueller matrix is modified, then the spectropolarimetric reconstruction would fail. However, if one assumes that the Mueller matrix is recalibrated post-fabrication, the spectropolarimetric reconstruction of the metasurface array can still be highly accurate.

To evaluate the spectropolarimetric reconstruction performance of metasurface arrays with fabrication errors, we used the recalibrated Mueller matrix to reconstruct a double-peak spectrum with varying peak separation in the simulation. The double-peak spectrum consists of two linearly polarized narrow-spectrum components. The first component has a fixed wavelength of 653.5 nm, while the second component has a tunable wavelength. Here, we assumed a Gaussian spectrum envelope. In the simulation, Gaussian noise with a signal-noise-ratio (SNR) of 10 dB was added to the signal. The spectropolarimetric reconstruction performance for metasurface arrays with different fabrication errors is shown in Fig. S9, with a spectral resolution of around 2 nm for all cases, which proves that the system is highly robust against fabrication errors, as long as the Mueller matrix of the metasurface array is calibrated post-fabrication.


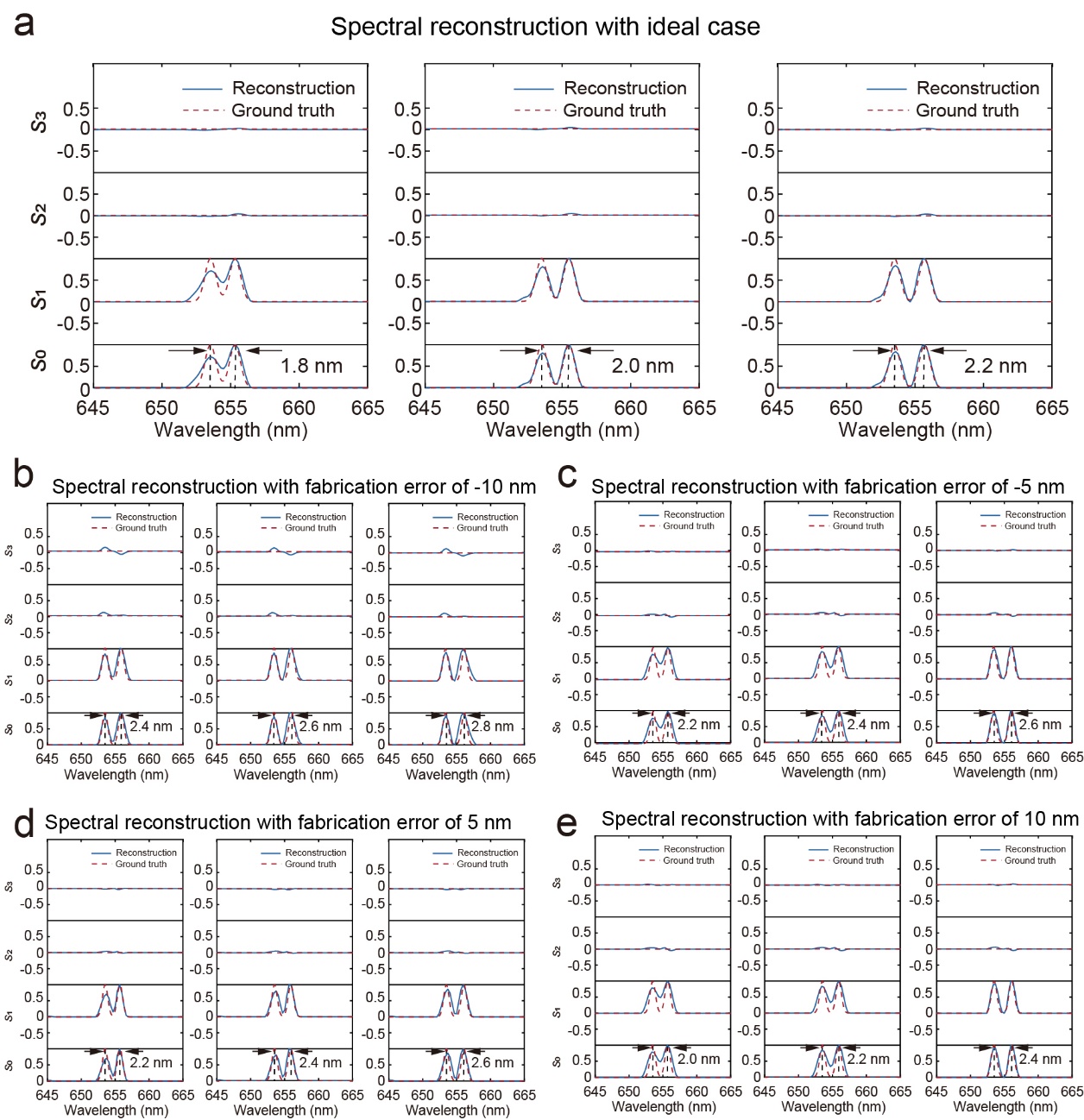


**Figure S9 | Analysis of the system's tolerance to fabrication errors.** **a-e,** Reconstructed double spectral peaks (blue solid line) of linearly polarized light for no fabrication error (a), fabrication errors of -10 nm (b), -5 nm (c), 5 nm (d), and 10 nm (e) compare with the ground truth generated by simulation (red dash line). The peak wavelengths are highlighted by the black dashed line.

**6. Theoretical resolution of metasurface array-based single-shot spectroscopic ellipsometry**

Theoretically, with a given Mueller matrix that is perfectly calibrated, the spectral resolution is dependent on the system noise level. As shown in Fig. S10a, we used the simulated Mueller matrix as the measurement matrix, assuming a system without noise, it can distinguish double spectral peaks separated by less than 1 nm (only limited by the spectral line width of the ground truth). With the SNR reduced to 15 dB (Fig. S10b) and 10 dB (Fig. S10c), the spectral resolution drops to 1.3 nm and 2 nm, respectively.

In the experiment, the spectral resolution, or the overall system performance, may be further affected by the deteriorated correlation of the measurement matrix (for example, due to fabrication errors) and by the inaccurate calibration of the Mueller matrix (for example, due to the imperfect phase retardation of the broadband quarter waveplate used during calibration).

**
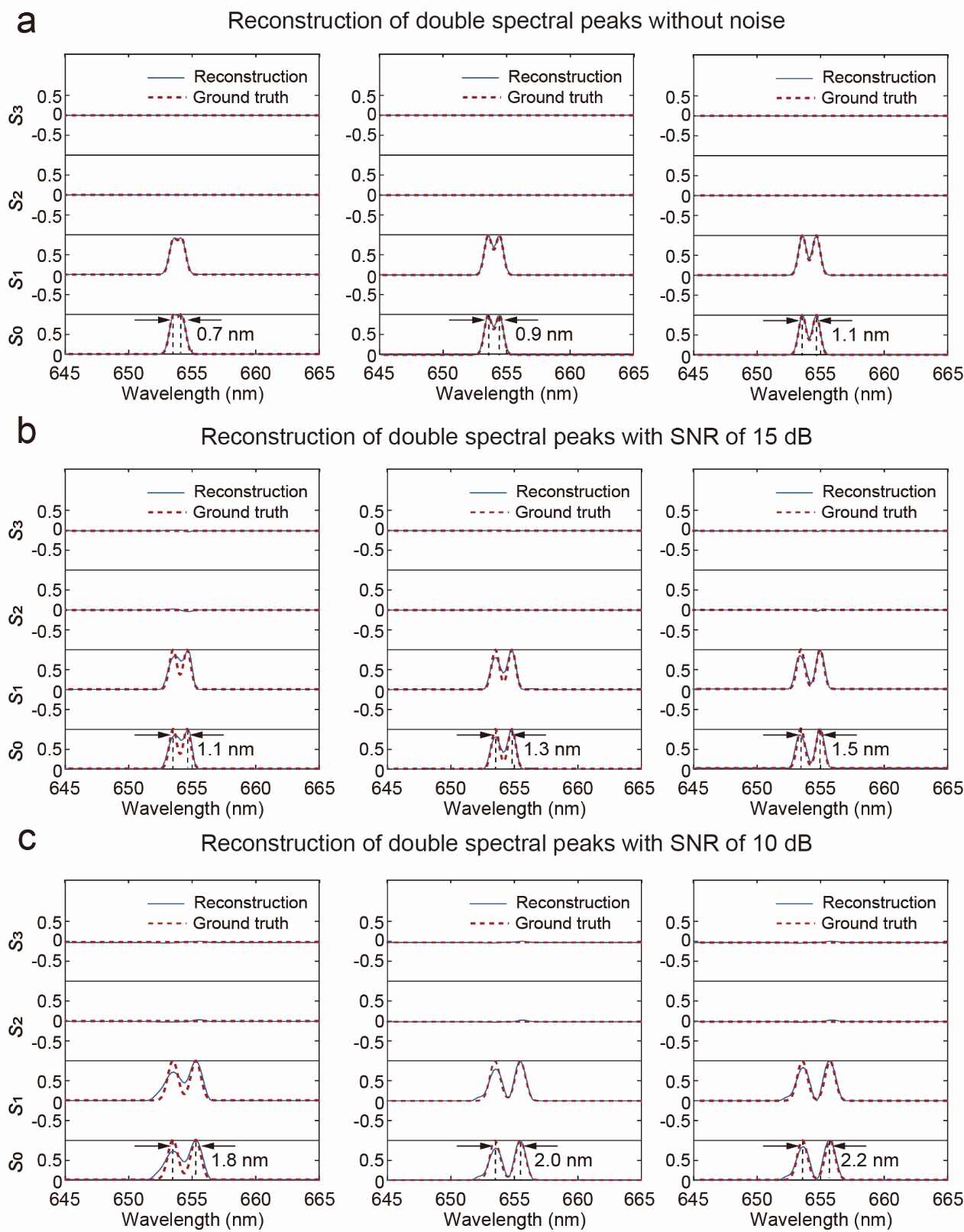
**

**Figure S10 | Reconstruction of double spectral peaks with different noise levels. a-c,** Reconstructed double spectral peaks (blue solid line) of linearly polarized light without noise (panel a), with SNR of 15 dB (panel b) and 10 dB (panel c), and compared with the ground truth generated by simulation (red dash line). The peak wavelengths are highlighted by the black dashed line.

**7.** **Reconstruction of multi-layer films with losses in simulation**

The ellipsometry measurement on a multi-layer film with losses is more challenging mainly for two reasons. First of all, for a multi-layer film, the reflected full-Stokes polarization spectrum typically has more complex spectral features, which makes the reconstruction more difficult. More importantly, the fitting model of a multi-layer film can be much more complex, with significantly more fitting parameters. Oftentimes, one needs to further consider issues such as surface roughness and inhomogeneity of the films.

Here, we did numerical simulations attempting to use a metasurface-array-based system for ellipsometry measurement of a bi-layer film consisting of a layer of lossy metal (Ag) and a layer of SiO_2_ sitting on a semi-infinite-thick Ag substrate. Here, the thickness of the Ag layer is assumed to be ranging from 15 nm to 25 nm, and the thickness of the SiO_2_ layer is assumed to be ranging from 1000 nm to 2000 nm. The refractive index of SiO_2_ is assumed to be constant of 1.457. The permittivity of Ag is assumed to follow the Drude model as:

$$\begin{aligned} \text{ε}_{\text{Ag}}\text{ = }\text{ε}_{\text{∞}} \text{- }\frac{\text{ω}_{\text{p}}^{\text{2}}}{\text{ω}\left( \text{1+}\text{iω}\text{ω}_{\text{τ}} \right)}\#\left( \text{S16} \right) \end{aligned}$$

where $\text{ε}_{\text{∞}}=\text{6}$ is the high frequency limit of the permittivity, $\text{ω}_{\text{p}}= \text{1.5 × 10}^{\text{16}}$ rad/s is the plasma frequency, and $\text{ω}_{\text{τ}}= \text{7.73 × 10}^{\text{13}}$ rad/s is the plasma damping rate ^5^.

The complex refractive index of Ag is calculated from the permittivity as:

$$\begin{aligned} \text{n}_{\text{Ag}}\text{ }\text{= }\text{n }\text{+ }\text{ik }\text{= }\sqrt{\text{ε}_{\text{Ag}}}\#\left( \text{S17} \right) \end{aligned}$$

where *n* is the real part of the complex refractive index, and *k* is the imaginary part of the complex refractive index.

With a plane wave incident at 60°, the full Stokes polarization spectra reflected by the bi-layer films with different thicknesses are simulated and shown in Fig. S11a-c, with the resulting ellipsometry parameters shown in Fig. S11d-f. The fitted complex refractive indices of Ag are shown in Fig. S11g-i, with the rest of the fitting parameters shown in Table S1, from which one can observe a close agreement between the fitted parameters and the ground truth.

|  | Film model 1 | | Film model 2 | | Film model 3 | |
| --- | --- | --- | --- | --- | --- | --- |
| Thickness | *d*_Ag_ | $\text{d}_{\text{SiO}_{\text{2}}}$ | *d*_Ag_ | $\text{d}_{\text{SiO}_{\text{2}}}$ | *d*_Ag_ | $\text{d}_{\text{SiO}_{\text{2}}}$ |
| Ground truth | 15 nm | 1200 nm | 20 nm | 2000 nm | 25 nm | 1000 nm |
| Reconstruction | 14.7 nm | 1182.4 nm | 20.1 nm | 1940.0 nm | 25.1 nm | 1015.4 nm |
| Refractive index | $\text{n}_{\text{SiO}_{\text{2}}}$ | | $\text{n}_{\text{SiO}_{\text{2}}}$ | | $\text{n}_{\text{SiO}_{\text{2}}}$ | |
| Ground truth | 1.457 | | 1.457 | | 1.457 | |
| Reconstruction | 1.471 | | 1.423 | | 1.487 | |

**Table S1 |** Comparison between the fitted film parameters and the ground truth for bi-layer films made of Ag and SiO_2_ with different thicknesses.


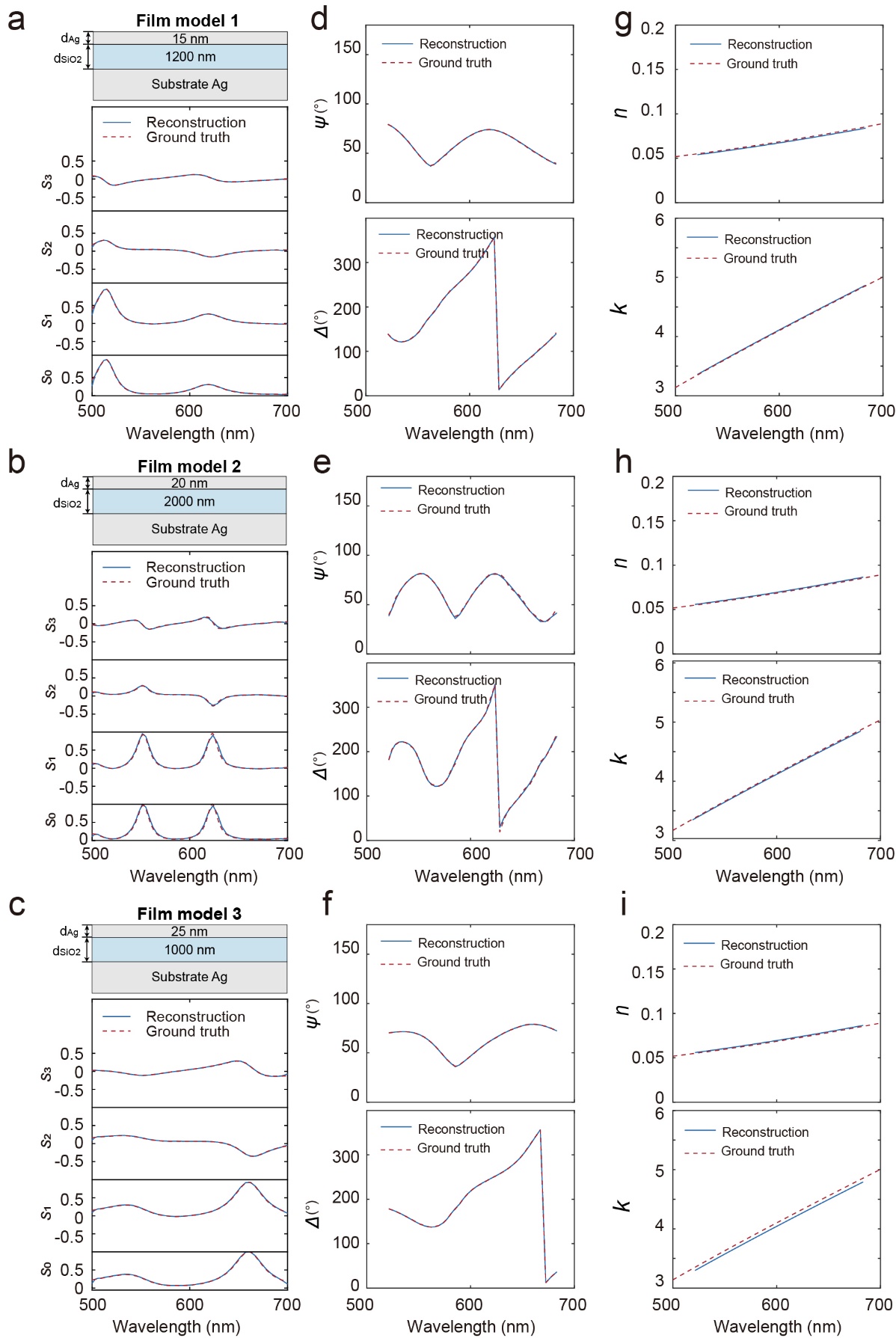


**Figure S11 | Numerically simulated spectroscopic ellipsometry reconstruction of bi-layer films made of Ag and SiO_2_. a**-**c**, Schematic of the film model and comparison between the reconstructed full Stokes polarization spectrum with simulated Mueller matrix (blue solid line) and the full Stokes polarization spectrum calculated by the multi-beam interference model (red dashed line). **d-f**, Comparison between the reconstructed ellipsometry parameters *Ψ* and *Δ* with simulated Mueller matrix (blue solid line) and ellipsometry parameters calculated by the multi-beam interference model (red dashed line). **g**-**i**, Comparison between the reconstructed refractive index of Ag with simulated Mueller matrix (blue solid line) and the ground truth refractive index of Ag calculated by Drude model (red dashed line).

**8. Metasurface fabrication process**

**
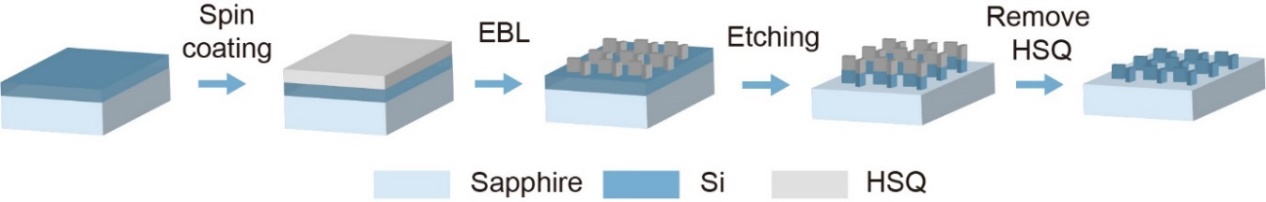
**

**Figure S12 |** Schematic of the fabrication process flow chart of the metasurface. EBL, Electron beam lithography; HSQ, hydrogen silsesquioxane, a negative-tone photoresist.

**9. Experimental setup for calibration**


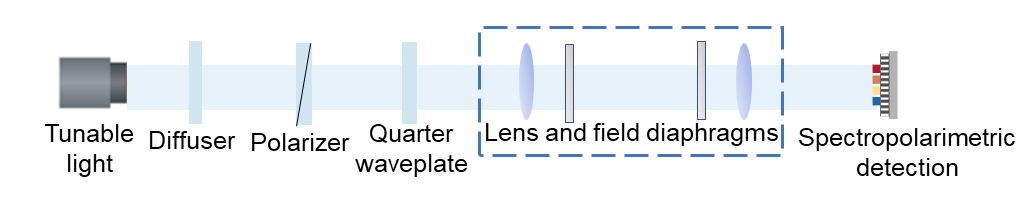


**Figure S13 |** Experimental setup for the calibration of ***M***_0_ of the metasurface array.

**10. The experimentally calibrated *M***_0_ **of the metasurface array.**

**
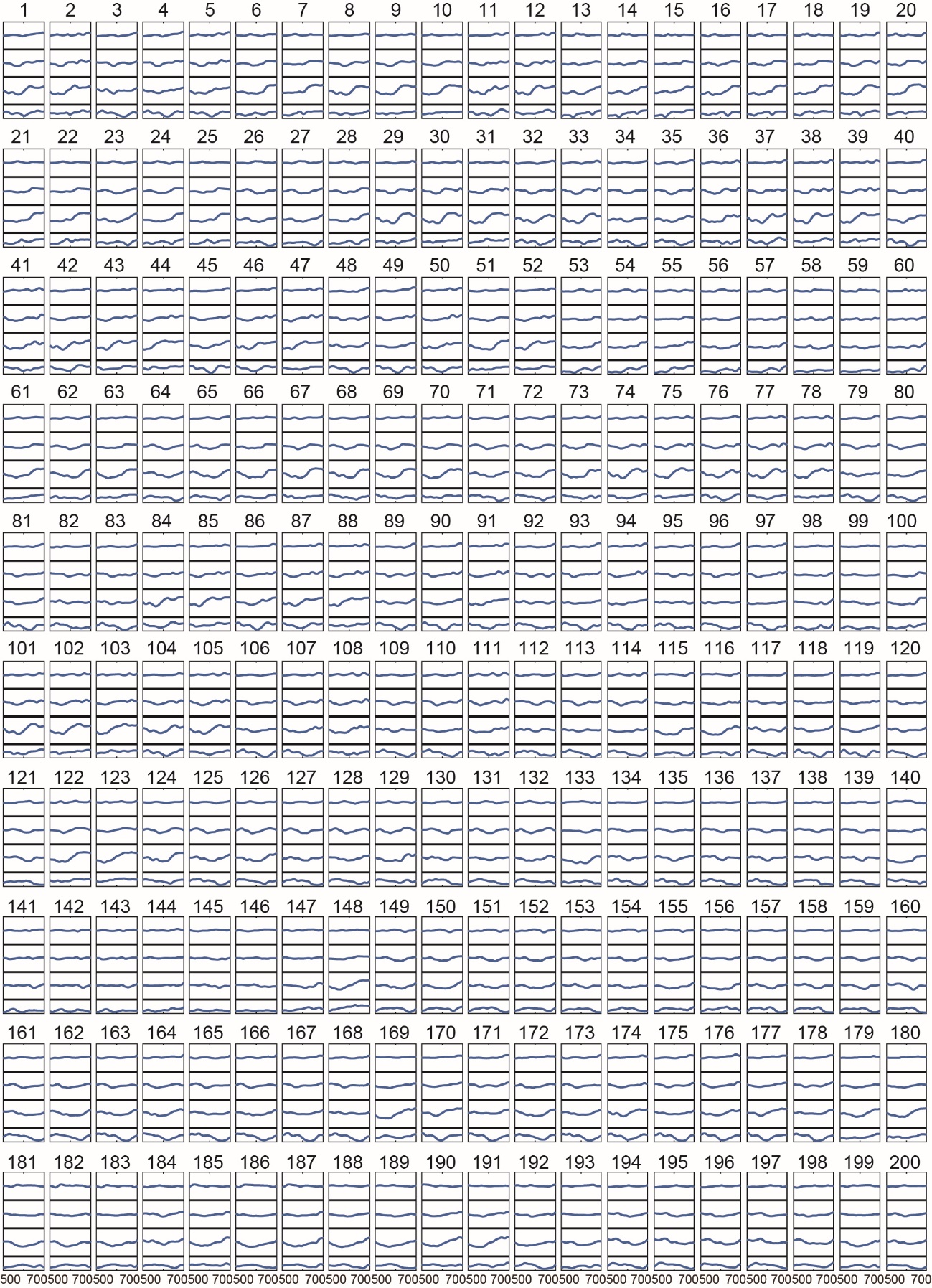
**

**Figure S14 | The experimentally calibrated** $\text{M}_{\text{0}}^{\text{i}}$ **of 1^st^ – 200^th^ metasurface elements.** In each subplot, the horizontal axis represents wavelength (in nanometers), and the vertical axis represents ***m***_00_, ***m***_01_, ***m***_02_, and ***m***_03_ from bottom to top, respectively.


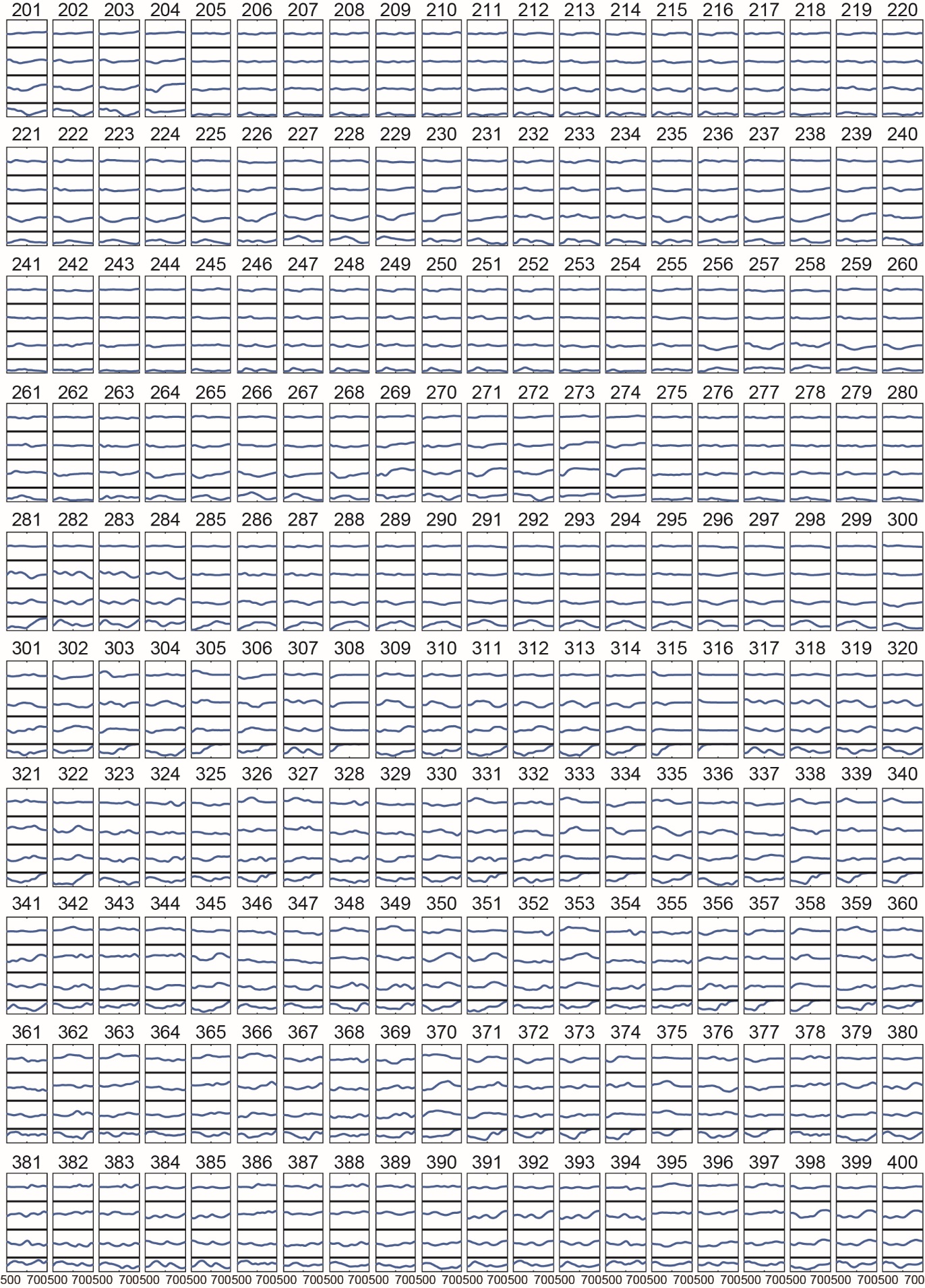


**Figure S15 | The calibrated** $\text{M}_{\text{0}}^{\text{i}}$ **of 201^st^ – 400^th^ metasurface elements.** In each subplot, the horizontal axis represents wavelength (in nanometers), and the vertical axis represents ***m***_00_, ***m***_01_, ***m***_02_, and ***m***_03_ from bottom to top, respectively.

**11.** **Spectropolarimetric reconstruction of dual-peak spectra**

**
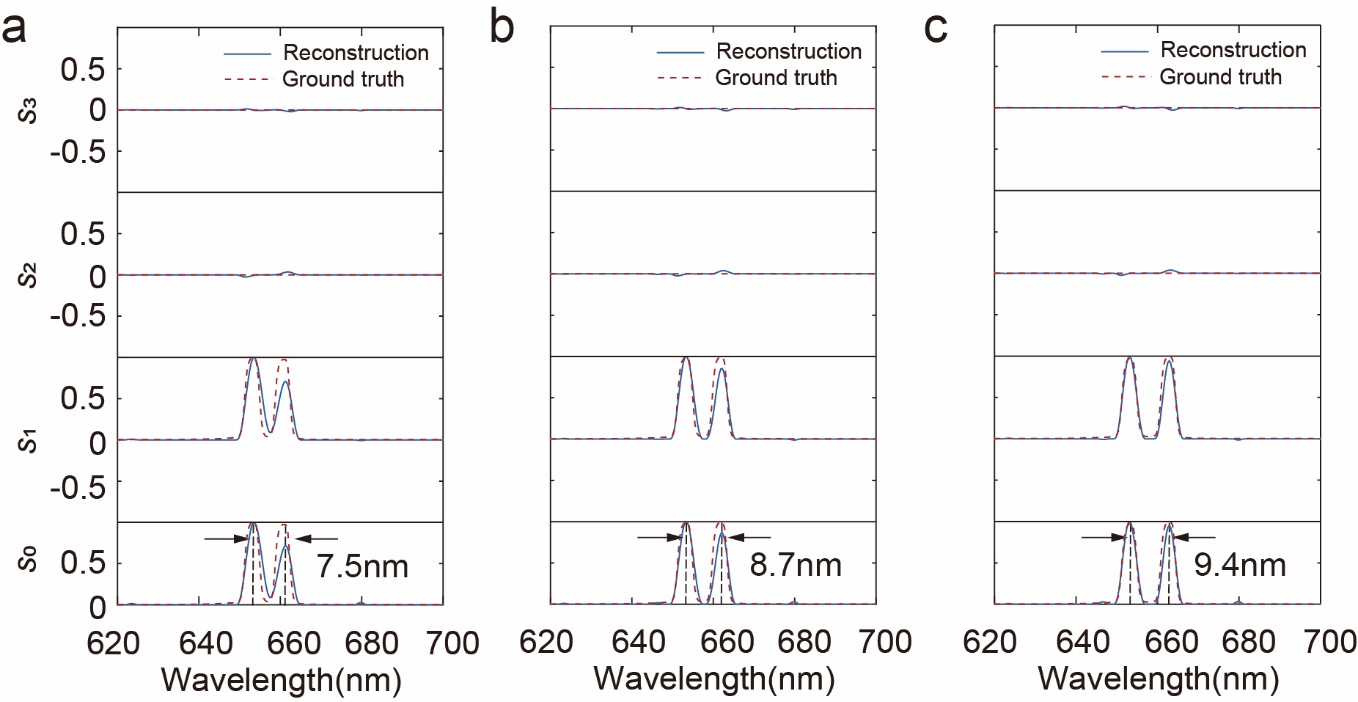
**

**Figure S16 | Characterization of the spectral resolution of the spectropolarimetric detection system.** **a-c**, Reconstructed full Stokes polarization spectrum of linearly polarized light with dual spectral peaks separated by 7.5 nm (a), 8.7 nm (b), and 9.4 nm (c), respectively, measured with the metasurface array-based spectropolarimetric detection system (blue solid line) compare with the ground truth measured with a quarter-waveplate, a rotating polarizer, and a conventional grating-based spectrometer (red dashed line). The peak wavelengths are highlighted by the black dashed line.

**12. Experimental setup for measuring the thin film properties**

**
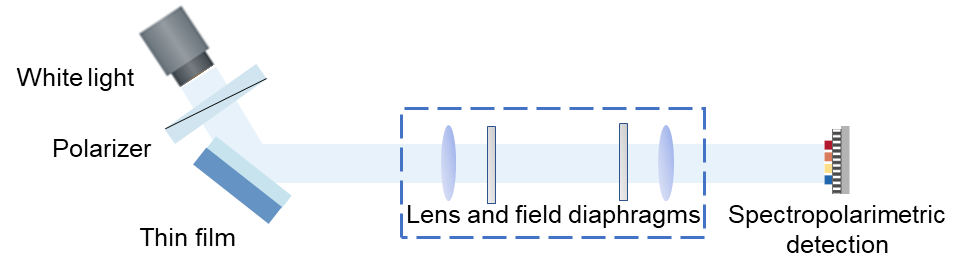
**

**Figure S17 |** Experimental setup for measuring the thin film properties.

**13. The reconstructed full Stokes polarization spectra and ellipsometry parameters of the SiO_2_ thin films.**


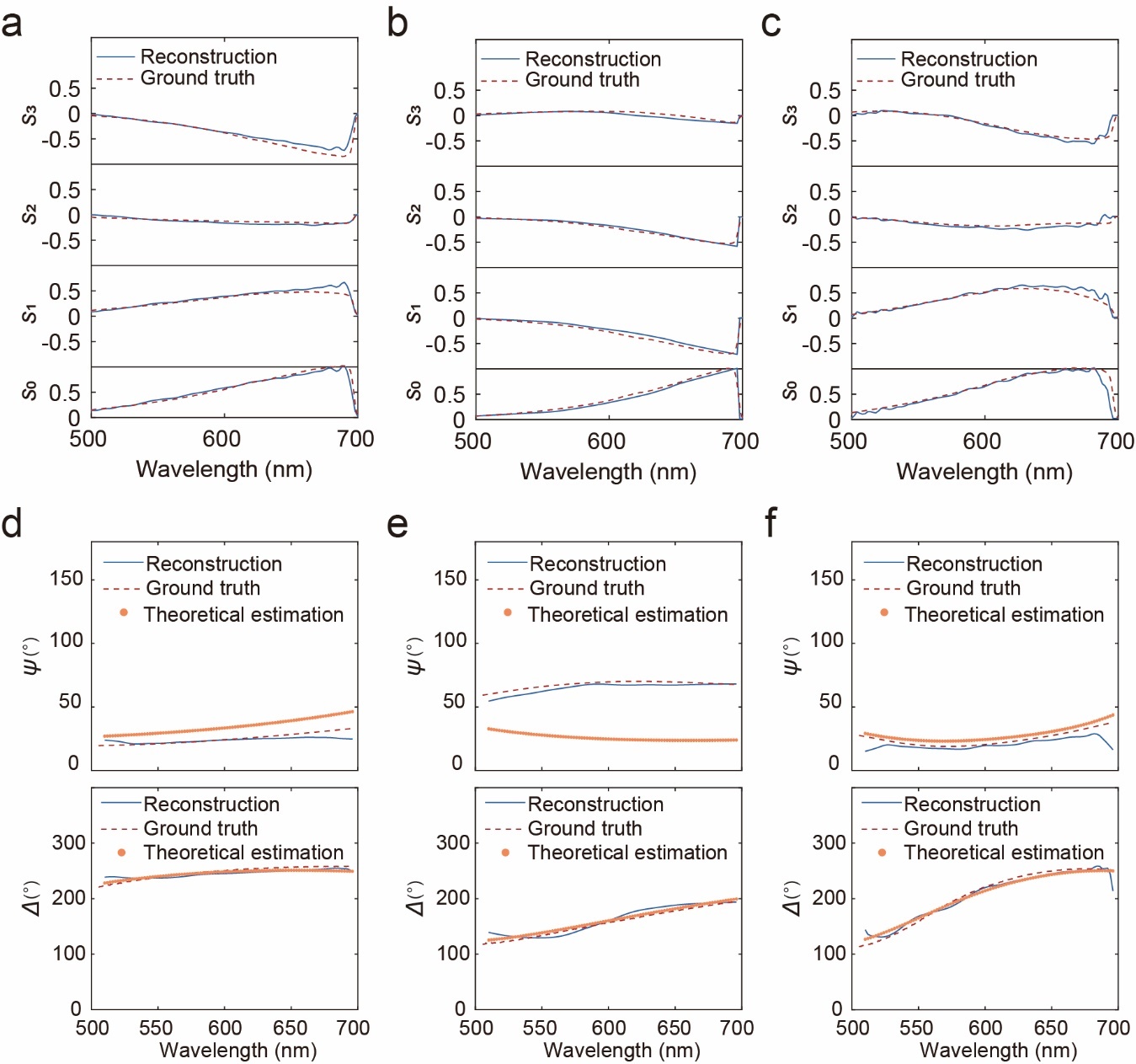


**Figure S18 | a-c,** Comparison between the reconstructed full Stokes polarization spectra from the single-shot spectroscopic ellipsometer (blue solid line), the ground truth (red dashed line) for the SiO_2_ thin film with a thickness of 200 nm (a), 300 nm (b) and 500 nm (c), respectively. The ground truth is measured with a quarter-waveplate, a rotating polarizer, and a conventional grating-based spectrometer. **d-f**, Comparison among the ellipsometry parameters *Ψ* and *Δ* from the single-shot spectroscopic ellipsometer (blue solid line), the ground truth (red dashed line) and the theoretical estimation (orange dots) for the SiO_2_ thin film with a thickness of 200 nm (d), 300 nm (e) and 500 nm (f), respectively. The ground truth is measured with a quarter-waveplate, a rotating polarizer, and a conventional grating-based spectrometer.

**14. The precision of the thickness and refractive index measurement for five SiO_2_ thin films.**

**
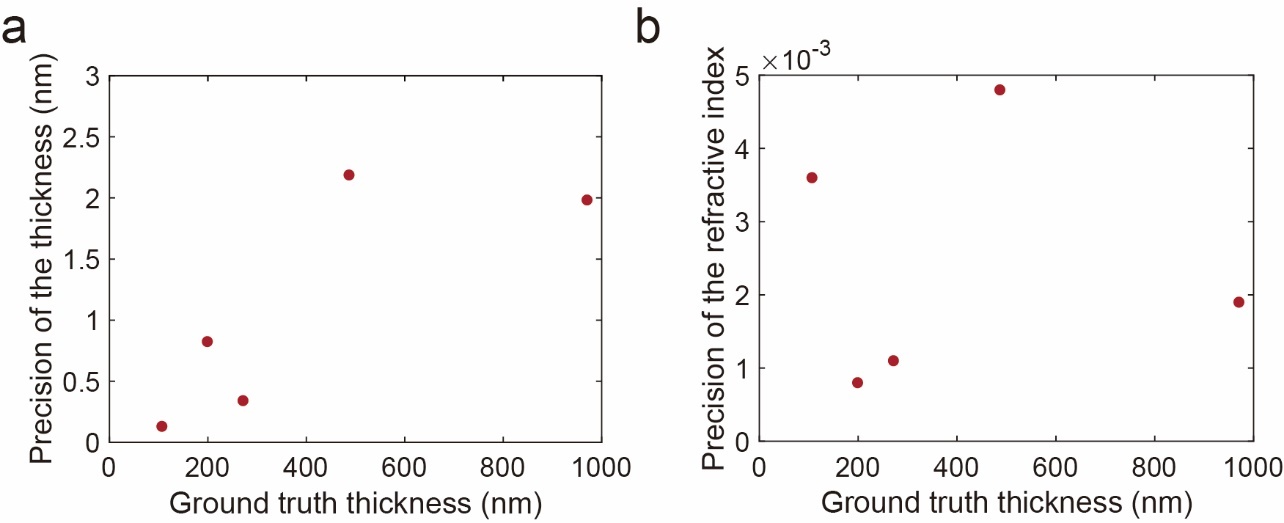
**

**Figure S19 | a**, Precision of the thickness measurement for five SiO_2_ thin films. **b**, Precision of the refractive index measurement for five SiO_2_ thin films.

**Supplementary Reference**

1 Ansys. Ansys lumerical FDTD. (2023). at <https://www.ansys.com/products/photonics/fdtd> URL.

2 Wang, S. et al. Arbitrary polarization conversion dichroism metasurfaces for all-in-one full Poincaré sphere polarizers. *Light: Science & Applications* **10**, 24 (2021).

3 Wu, C. et al. Spectrally selective chiral silicon metasurfaces based on infrared Fano resonances. *Nature Communications* **5**, 3892 (2014).

4 Palik, E. D. Handbook of Optical Constants of Solids. (Boston: Academic Press, 1997), xiii-xv.

5 Liu, Z. W. et al. Tuning the focus of a plasmonic lens by the incident angle. *Applied Physics Letters* **88**, 171108 (2006).
